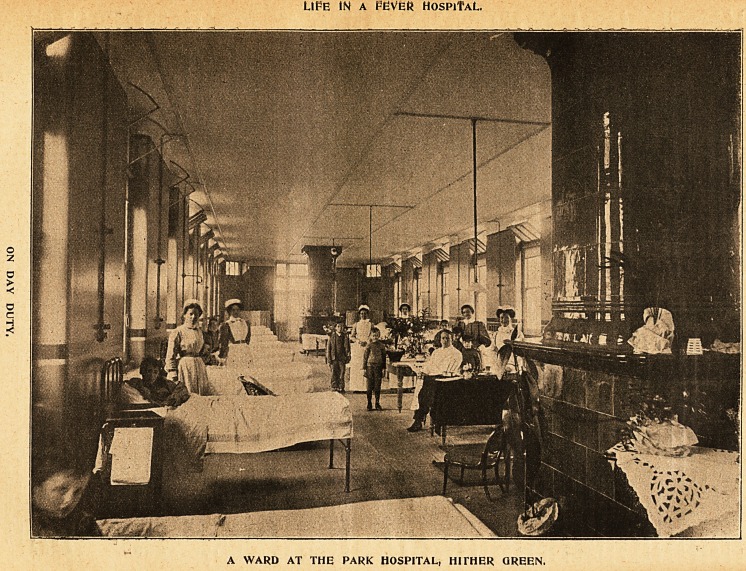# The Hospital. Nursing Section

**Published:** 1907-02-16

**Authors:** 


### The Hospital.

### Nursing Section.

Contributions for "THE HOSPITAL," should be addressed to the EDITOR, "THE HOSPITAL" NURSING SECTION, 28 & 29 Southampton Street, Strand, London, W.C.

No. 1,066.—VOL. XLI. SATURDAY, FEBRUARY 16, 1907.

### Notes on News from the Nursing World.

### DEATH OF THE COUNTESS CADOGAN.

NURSES in many parts of the country will share our deep regret at the death of Countess Cadogan, which took place on Saturday last. Her interest in nursing and sympathy for nurses was shown in a variety of ways. The extension of the Jubilee Institute to Ireland was mainly due to her efforts, and when Queen Victoria visited the sister island, she arranged that the Queen's nurses should have the pleasure of being inspected at the Viceregal Lodge by her Majesty. Subsequently, they enjoyed her hospitality. Lady Cadogan was for some years a member of the Advisory Board of the Junius S. Morgan Benevolent Fund of the Royal National Pension Fund for Nurses, and her services in this capacity were highly appreciated by her colleagues.

### THE RESIGNATIONS AT THE BRITISH LYING-IN-HOSPITAL.

AT a meeting of the Board of Management of the British Lying-in Hospital on Tuesday last the resignations of the matron, Miss Gertrude Knott, and of the sister, Miss Alice Sanderson, were presumably discussed, and it is understood that much regret was expressed that they had been tendered. The work carried on under the auspices of Miss Knott and her colleague were so fully described in our issue of January 26 that nothing remains to be said concerning it. The matron, in the interview with our Commissioner, did not complain of the fact that no resident medical officer is attached to the hospital; but there is no doubt that she has felt the need of such an appointment, and that she has at last rebelled against a state of affairs which had become intolerable. Both Miss Knott and Miss Sanderson will leave the institution in Endell Street in April.

### THE CATERING AT A MILITARY HOSPITAL.

THE sisters at the Colchester Military Hospital have been complaining through the medium of *Truth* that the catering is unsatisfactory. They are allowed 13s. a week for mess money and they think that the matron of the hospital does not spend this sum to the best advantage. *Truth* thereupon suggests that the messing should be supervised by a committee such as exists in connection with every other Service mess. We do not concur in this proposal. The matron of a hospital is the proper person to cater for the nurses, and how admirably fitted she is in some cases for this duty is shown by an article on another page in which the matron of a civil provincial hospital sets forth her experiences. It is impossible to criticise the Colchester matron's talents for catering without knowing how far she is hampered by circumstances outside her control. Excellent diet should be forthcoming on the very liberal allowance quoted; nay, even on half that sum. The simple process of engaging a new cook and changing the butcher would probably work wonders.

### CHARGE NURSES AND FEVER TRAINING.

WE are glad that the Irish Local Government Board stick to their guns, and, after the fullest consideration, have refused to alter the qualifications for the position of charge nurses, which therefore will now always include six months' training in a fever hospital. With reference to the contention of the medical officer at the Lurgan Fever Hospital that four months and a half in that institution should be considered sufficient, the Local Government Board are unable to reconcile the statement of the medical officer, that each probationer attended from fifty to sixty cases in four months, with the fact that less than thirty cases were treated in the hospital during six months. In any event, the Lurgan Guardians, we presume, will recognise that in the interests of their training school the determination of the Local Government Board must be respected.

### TIDING OVER THINGS AT KING'S LYNN.

THE Mayor of King's Lynn announced at the last meeting of the Guardians that two nurses had been engaged for a time, "owing to the exceptionally large number of bad cases in the infirmary." But the Rev. A. H. Hayes, who thanked the Mayor for taking such a hopeful view of things, said it was singular that the exceptional circumstances had lasted more or less since the staff was reduced last August; and said that the wisest policy was to be always ready for any emergency. We agree with him; the tiding-over policy, which finds favour with a majority of the Lynn Guardians, is generally not only inefficient, but in the long run extravagant.

### A MANCHESTER MATERNITY HOME.

AT an inquest in Manchester the other day on the body of an infant found dead in a maternity home in that city the Coroner remarked that two years ago there was a similar case in the home. In this instance the mother took the child from its cot in the night, and the cause of death was asphyxia. It was stated, in the course of the evidence, that the staff at the home consisted of two nurses and a probationer, and that there was no nurse on duty during the night. The jury recommended that a night nurse should be provided. Compliance with this recommendation will obviate such dangers in the future, but the second warning should not have been required. A maternity home without a night nurse is a survival of slipshod organisation which must no longer be tolerated anywhere.

### THE CASE OF A SUPERINTENDENT NURSE.

It is a very unusual thing for an official who is working under the Local Government Board to persist in retaining a position which cannot be held with advantage when the presentation of a report by its inspectors is followed by an intimation from Whitehall that the post must be resigned. In the case of the superintendent nurse of the workhouse of the Auckland Union, the central authorities, while refusing to furnish her with extracts from the report submitted to them, made it clear that there is no slur on her character by expressing their readiness to sanction her appointment to the workhouse of some other union. We do not understand why, in these circumstances, the Auckland Guardians decline to give her notice, and have called upon the Local Government Board to do so.

### THE INFIRMARY AND THE NURSING SOCIETY.

At the annual meeting of the Perth Sick Poor Nursing Society, which was held the other day, considerable astonishment was expressed that the directors of Perth Royal Infirmary have intimated their intention not to continue the contribution of £60, which they have annually made to the funds of the Society for the last twelve years for work done by the latter in nursing the outdoor patients of the infirmary. The Chairman of the Society, speaking as one of the large subscribers to the infirmary, said that he was somewhat surprised at the action of the directors of that institution, in suddenly cutting off their support from an Association which he might almost call a limb of the infirmary. But there was a pleasant note of confidence at the meeting that if the infirmary directors adhere to their determination, the public will make up the deficiency caused by the withdrawal of the grant. It was, of course, maintained that in such circumstances the society cannot afford to treat any of the discharged infirmary patients. Perhaps there is room for a compromise.

### THE INSPECTION OF SCHOOL CHILDREN.

At the annual meeting last month of the Garston and District Nursing Association, lately affiliated to the Jubilee Institute, mention was made of the anxiety of the committee to secure a second nurse, who would be able to visit each school in Garston at least one day in the week, for the purpose of giving attention to, and reporting upon, the ailments of the children. Miss Amy Hughes, who was present at the meeting, having referred to the Garston Association as one of the oldest established in the country, said that all concerned would congratulate themselves upon the way in which the work was carried on. She emphasised the importance of children being inspected at school, and observed that "Liverpool has a leading position in regard to this special kind of work." We are glad to see that greater Liverpool, of which Garston forms an important part, seems determined to maintain that position.

### POOR LAW PROBATIONERS AND SALARIES.

At a meeting of the Colchester Guardians a recommendation of the Infirmary Committee that the salary of an assistant nurse should be raised from £10 to £12 10s. at once, and from £12 10s. to £15 next year, was discussed at some length. The Chairman of the Infirmary Committee opposed the proposal on the ground that it looked like increasing the nursing expenses by a side wind, and another Guardian said that he did not think that the system of paying probationers a stated salary should be departed from. On the other hand, it was maintained that the probationer is a good "lifter," and is at present doing as much work as a nurse at a salary of £25; and in the end the increase was agreed to. In ordinary circumstances the salary of a probationer is, of course, fixed for the term she is serving, but at Colchester they are not engaged for a definite period. This being so, the Guardians were no doubt well advised in assenting to the recommendation of their Infirmary Committee; but it is, of course, far better to have a given time of training and a fixed rate of remuneration.

### DISTRICT NURSES FOR NIGHT DUTY.

It is a very high compliment indeed to say of an organisation that its management is not capable of improvement. This, however, is the testimony given by Dr. Handcock at the annual meeting of the Bradford District Nursing Association, and it is the more noteworthy because Dr. Handcock has more opportunities than most people in Bradford of seeing how valuable the work of the nurses is in the homes of the poor. He thinks, however, that, while he could propose no improvement in the management, the institution is capable of extension; and he suggests that the provision of an extra nurse for night work would be a great boon. Under present circumstances, the poor people in Bradford who are taken urgently ill at night cannot get help. It is obvious that a district nurse, of all nurses, cannot work during the night as well as in the day, and we look forward to the time when not only Bradford, but many other District Associations will be able to afford to detail a nurse off for night duty.

### MIDWIFERY IN LINCOLNSHIRE.

A feature in connection with the operations of the Lincolnshire Nursing Association, whose annual meeting took place last Friday, with the Duke of Rutland as chairman, is that the importance of midwifery training is being increasingly recognised. When the Midwives Registration Act first came into operation the Chairman of the Lindsey County Council stated that of the ninety-one midwives known to be practising in Lincolnshire, only seven were registered. That number, we learn from the report of the Nursing Association, has now been almost quadrupled, and at the present time twenty-seven are registered, while the Nursing Association and the County Council are as quickly as possible training more nurses to receive the midwifery certificate. It is obvious that there is still a great deal of room for improvement, but we rejoice to observe that the managers of the county nursing organisation are taking up the work of augmenting the supply of trained midwives both sympathetically and systematically.

### MISSION NURSES AND LITTLE HELPERS.

A PLEASANT little gathering was held the other day at the headquarters of the Biblewomen and Nurses' Mission, which has started a branch of juvenile workers under the name of "Little Helpers of the Poor." All the members of this Children's League living in London were invited. They were received by Mary Countess of Harrowby. Miss Andrews, the Hon. Superintendent, spoke of the work and aims of the League, and showed how much can be done towards helping and cheering the thousands of sick children to whom the Mission nurses go by means of a little unselfish thought, sympathy, and small sacrifices. Each member is expected to subscribe 6d. a year, and to fulfil one or two other simple conditions.

### INCREASE OF A MATRON'S SALARY.

AT the last meeting of the governing body of Radcliffe Infirmary, Oxford, the Treasurer, as Chairman of the Committee of Management, moved that the salary of the matron be increased from £120 to £140 a year. For nine years, he said, the present matron, Miss Wait, had been invaluable in the conduct of the place, and he did not think, if he looked all round Oxford, he could find a person who was more highly appreciated in the duties she discharged. The Rev. W. W. Merry seconded the motion, and after Mrs. Green had observed that they owed much to Miss Wait's tact, wisdom, knowledge, conduct of the nursing and of the domestic part of the infirmary, it was unanimously agreed to. We congratulate the matron upon the compliment paid to her, and all the more so because the Treasurer expressed his regret that the state of the finances prevented him from proposing a larger addition.

### THE LATE SUPERINTENDENT OF THE LINCOLN INSTITUTION OF NURSES.

THE death of Miss Henrietta Bromhead, lady superintendent of the Lincoln Institution of Nurses, at the age of 59, deprives the nursing world in that part of the country of a conspicuous figure. Miss Bromhead, whose funeral took place on Tuesday last week, the service being held in Lincoln Cathedral, devoted 20 years of her life to the work which her mother originated in 1866. How largely it has developed may be gathered from the fact that the fortieth annual report shows that there are now 70 nurses on the staff of the institution. Our readers will remember that at the time of the epidemic of typhoid fever in Lincoln, two years ago, the nursing was placed entirely in the hands of Miss Bromhead. The account which appeared in our columns during the epidemic was written by her, at our request. In consenting, she expressed her desire that no prominence should be given to her own efforts, which, thanks to the hearty cooperation of her capable staff, were attended by remarkable success. The memorial of Lincoln to Miss Bromhead is already in existence. In the Jubilee year no less than £5,000 was collected in Lincoln in celebration of the reign of Queen Victoria and invested, the interest—about £140—being assigned to the superintendent of the nursing institution to be used during her lifetime in aid of the district nurses.

### A PIONEER OF NURSING.

THE death of Miss Jane Bowen, sister of Bishop Bowen, of Sierra Leone, at the age of 83, in the little village of Little Haven, merits mention in our columns, because it was through her efforts that the first Queen's nurse appointed after the Jubilee gift of Queen Victoria was sent to St. Bride's in that remote part of Pembrokeshire. For many years Miss Bowen herself acted as doctor, dispenser, and nurse, and the nearest medical aid is still seven miles off. But the presence of a Queen's nurse in the district has made a great difference, and is immensely appreciated.

### THE MATRON OF THE BOLINGBROKE HOSPITAL.

WE are glad to hear that the matron of the Bolingbroke Hospital, Wandsworth Common, returned to her duties this week. Miss Russell has been recuperating at Hastings after her serious illness, and is now looking quite fit and well again. The nursing staff were extremely pleased to welcome her back.

### DISTRICT NURSES AND THE DEATH-RATE.

AT the annual meeting of the Lancaster Nursing Society this month, Mr. Helme, M.P., was one of the speakers. In proposing the re-election of the president, Mrs. H. L. Storey, he said that he did not think he was going too far to say that in Lancaster they owed the improved death-rate to the work of the district nurses in the homes of the poor. This is very valuable testimony of the Society's value to the town, and we are glad to learn that the financial condition of the Association is satisfactory. Both the subscriptions and the Church collections showed an increase last year, and the slight augmentation of expenditure was due to a rise in the salaries of the nurses in accordance with the rules. The number of cases nursed was 462, of visits paid 12,396, and the average cost of each visit 6½d. The number of visits paid per day by the nurses was 35.

### ENTERTAINMENT TO BELFAST NURSES.

LAST week the nurses of the Belfast Union Infirmary were entertained by a number of friends, the entertainment commencing with a concert, which was held in the recreation-room of the Nurses' Home. Miss Ward, the superintendent of the nurses, rendered willing assistance and facilitated in every way the carrying out of an excellent programme. Subsequently the nurses and numerous visitors had a social reunion in the dining-room.

### SHORT ITEMS.

LADY HERMIONE BLACKWOOD, President of the Belfast branch of the Irish Nurses' Association, gave an entertainment on the 4th inst., when she was "At Home" from 7.30 P.M. to 10 P.M.—A course of six lectures on "The Hygiene of Child Life" will be given by Miss M. Const. Barker, in the Lecture Room, 53 Berners Street, W., on Wednesdays, February 20 and 27, and March 6, 13, 20, and 27, 1907, at 2.30 P.M., under the auspices of the National Health Society.

### The Nursing Outlook.

"From magnanimity, all fears above;
From nobler recompense, above applause,
Which owes to man's short outlook all its charm."

### MAKE-BELIEVE IN BRITISH NURSING.

### I. THE STAGE ARMY AND ITS MACHINERY.

It is announced that in June next there will be a conference held in Paris of the International Council of Nurses. It may be remembered that the last conference of the kind was held at Berlin, when there were present some thirty nurses from Great Britain, of whom only one, Miss Isla Stewart, was matron of a large London hospital, the other British delegates holding such positions as the superintendent of Sir Patrick Dun's Hospital, the matron of the General Hospital, Birmingham, the matron of Much Wenlock Hospital, the Queen's nurse from Surbiton, and a sister from the Royal Hants Hospital. The United States had a somewhat similar representation; Germany supplied a dozen members, and there were single delegates from France, Canada, Denmark, Holland, and Sweden. It may be interesting to examine the causes of the small attendance of Bristol nurses at Berlin in 1904.

An instructive sidelight on the hollowness of the machinery upon which this International Council rests, so far as Great Britain is concerned, is supplied by the publication of what purports to be the annual report of the Matrons' Council, submitted to a meeting held on January 31st, to which reporters were not invited. This document exhibits the reasons why no doubt publicity is not sought for the proceedings of this body, why no list of its members can be obtained, and why British nursing is made to look so ridiculous in the eyes of other countries through the folly of a few ambitious spirits, whose prime movers are mostly ex-matrons, that is, ladies who have retired from active participation in the education and training of nurses. It is noticeable that the ambitious spirits in question display exceeding modesty so far as publicity is concerned, when they hold a meeting of the so-called Matrons' Council, on which, it will be seen, the whole superstructure of the British end of the International Council and of the National Council of Nurses in reality rests.

Who then are the Matrons' Council? The only information forthcoming on this point is to be found in an advertisement which has been running in practically the same form for some thirteen years. The advertisement contains the names of Miss Isla Stewart, the president, of three ex-matrons, including Mrs. Bedford Fenwick, and of the matron of the General Hospital, Nottingham, the Royal Hants Hospital, Southampton, and the Infirmary, Leicester. The Honorary Secretary is Miss M. Breay, and the report indicates that Mrs. Bedford Fenwick and Miss Breay in fact supply most of the initiative and do most of the work. No report or statement of accounts appears to be published by the Matrons' Council, but its financial position is indicated by a statement that, the balance in hand at the moment is under £9. In the report referred to we get a clear description of the stage army and its proceedings. Thus, the Matrons' Council, on July 1st, 1899, on Mrs. Bedford Fenwick's initiative, resolved to organise an International Council of Nurses. In 1900 the constitution drafted by these ladies was adopted, and Mrs. Bedford Fenwick was elected first President of the Council. The first meeting of this International Council (?) was held in Buffalo in 1901, the second in Berlin in 1904, and the third is to be held in Paris in June next. On October 26th, 1899, on the motion of Mrs. Fenwick, the Matrons' Council decided to form a provisional committee to organise a National Council of Nurses, such provisional committee consisting of the executive of the Matrons' Council. The Executive relegated the matter to a sub-committee consisting of Miss Isla Stewart, Mrs. Fenwick, and Miss Breay. At the annual meeting of the Matrons' Council in 1900 the report of this sub-committee was under consideration, together with a draft constitution of the so-called National Council of Nurses. The National Council idea would appear to have then halted in its progress until 1904. In the intervening years the Matrons' Council endeavoured to organise Leagues of certificated nurses in connection with their training schools, and ultimately Miss Isla Stewart summoned a conference of delegates of such leagues, which recommended the constitution of a National Council, when 5,000 nurses were represented by delegation. On November 25th, 1904, before apparently the National Council could be constituted on the basis recommended, for the reason that delegation by 5,000 nurses could not be secured, the provisional committee affiliated itself with the International Council of Nurses. It is therefore doubtful whether at the moment England is blessed with a National Council of Nurses, or whether it is merely a provisional committee of the Matrons' Council which has affiliated itself with the International Council. In 1902 the Matrons' Council instituted the Society for the State Registration of Trained Nurses.

It would thus appear that the Matrons' Council besides other things, is, for practical purposes, the National Council of Nurses, the Society for the State Registration of Trained Nurses, and the British Section of the International Council of Nurses. That is to say, these eight ladies—some of whom are not now actively engaged in nursing—calling themselves the Matrons' Council, constitute, in fact, the bone and sinew of all these high-sounding Councils and Societies. Could make-believe in nursing be more humorously exhibited for the laughter of the whole profession throughout the world?

### The Care and Nursing of the Insane.

By PERCY J. BAILY, M.B., C.M.Edin., Medical Superintendent of Hanwell Asylum.

### II.—NURSING THE SICK.

(*Continued from page* 263.)

*Enema.*—Any liquid preparation which is introduced into the bowel is called an enema. The purposes for which an enema may be ordered are very various, and the form and size of the injection and the apparatus required for its introduction into the bowel vary with the purpose for which it is used. There are three chief varieties of enema, which are: (1) purgative; (2) medicinal; (3) nutritive.

(1) *Purgative enema.*—The object of these is to clear the bowel of its contents. The commonest kind of purgative enema consists of a large amount of fluid—from one to two pints, or even more—which is injected into the bowel by means of a Higginson's syringe. In some cases, when very large quantities of fluid—as much as two quarts are sometimes given—are to be used it is convenient to have an india-rubber tube (a No. 12 catheter) attached to the nozzle of the syringe. The position of the patient should be the same as already described for the administration of a suppository, but the buttocks should be raised on a pillow and the head should be low. Over the pillow on which the buttocks rest a mackintosh sheet covered with a draw-sheet should be arranged in order to protect the bed. All the bed-clothes should be turned back so as to be out of the way, the patient being covered with a blanket. The enema should be prepared in a deep basin so that the end of the tube of the Higginson's syringe may be always covered by fluid; and must be heated to near the temperature of the body—about 95° F. The temperature must be tested with a thermometer. The basin containing the enema should be placed in a convenient position on a small table or stool near the side of the bed. The nozzle of the syringe or the india-rubber tube, if one be used, should be oiled and the syringe filled with the fluid, whatever it may be, so that there may be no fear of pumping a syringe full of air into the bowel before the enema. The nurse standing at the patient's back must then pass the index finger between the patient's buttocks until the anal orifice is found, and with the right hand insert the nozzle or tube into the rectum. The first reflex contraction of the sphincter which always takes place as soon as the tube reaches the anus very soon relaxes, and the tube will then readily enter. It should be remembered that the direction of the bowel is not directly upwards from the anus, but inclines rather backwards and towards the left, and that no real force should be necessary to make the tube pass through the anus. Special care should be exercised by the nurse during this part of the operation when an india-rubber tube is used, and which is intended to enter the bowel to the extent of some six or eight inches, for if any great force be employed there is a risk of piercing the wall of the rectum, and of subsequently pumping the enema into the peritoneal cavity—an accident which would inevitably set up fatal peritonitis. When the nozzle has been satisfactorily inserted the enema is slowly and steadily pumped into the bowel. If this part of the operation is performed too quickly there is a danger of its being at once expelled, and the object of the enema thus frustrated. The time occupied should be from three to five minutes to each pint. When the whole of the enema has been passed into the rectum the tube must be gently withdrawn, and a warmed towel should then be pressed against the anus for a few minutes. The object of doing this is to help to overcome the almost irresistible desire the patient experiences to expel the enema at once. It should be retained in the bowel for 10 or 15 minutes if possible, when the patient, if he is able to get out of bed, should be placed on the night stool. If he is unable to be removed from the bed a bed-pan must be placed under him.

After the administration of a copious enema there is a danger of the patient fainting, and the nurse should be prepared for such an eventuality.

The ordinary purgative enema for use in cases of constipation should consist of two pints of soap and water for an adult; for a child of four or five years from four to six or seven ounces may be used. To this may be added for an adult three or four ounces of turpentine—or very thin gruel may be used either alone or mixed with from three to eight ounces of olive oil, or with two ounces of castor oil, or the basis of the enema may consist of thin starch.

Small quantities—one or two drachms—of glycerine may be injected into the rectum to produce a purgative effect. For this purpose a special form of syringe, usually made of vulcanite, is necessary. It produces a natural motion in about half an hour. Glycerine is apt to give rise to some discomfort, amounting in some cases to a severe burning sensation.

(2) *Medicinal Enemata.*—These may be used for a variety of objects, the amount and the composition of the enema depending upon the effect which is desired. Usually, but not invariably, medicinal enemata are used in order to produce some local effect upon the bowel.

The chief varieties of this class of enema are the following:—

(a) *Sedative.*—These are given for the purpose of relieving pain or spasm in cases of severe diarrhœa, dysentery, or other painful conditions of the lower bowel. This kind of enema is intended to be retained in the bowel, and is therefore always small in amount. The best example is the starch and opium enema (enema opii) of the British Pharmacopœia. The best apparatus to administer it with is an ordinary two-ounce glass syringe, to the nozzle of which is attached a few inches of a No. 12 rubber catheter.

(b) *Astringent.*—These enemas consist of solution of various metallic salts, such as nitrate of silver (5 grains to a pint), sulphate of zinc or sulphate of copper (1 grain to an ounce). Naturally the choice of these does not lie with the nurse, but she should have an intelligent interest in their composition and uses. They are given usually in comparatively large quantities, and the rules for their administration are the same as those for an ordinary purgative enema. Dysentery is the chief disease in the treatment of which they are ordered.

(c) *Enemata for destroying intestinal worms.*—The common thread worm is the parasite most readily treated by means of rectal injections, which may consist of a solution of table salt. Two teaspoonfuls to half a pint of water—or spirit of turpentine ʒij to ʒiij mixed with the yolk of an egg in ℥iv. of water or infusion of quassia ℥j to half pint of water. These injections are best given with an ordinary Higginson's syringe, or with the apparatus which will shortly be described, when dealing with the nutrient enemata. In every case, before the enema is administered, the preparatory treatment ordered by the medical attendant must be carefully carried out. This consists in the administration of an ordinary purgative enema or a dose of castor oil or other purgative, so as to clear the lower bowel of any fæcal accumulation. The patient must always be directed to retain the enema as long as possible, and for this reason the amount given is never to be very large—usually about half a pint—never more than one pint.

(d) In the treatment of epilepsy, when the patient passes into what is known as the status epilepticus—that is to say, when the fits follow one another so rapidly that there is no appreciable interval between them—an enema consisting of a solution of chloral hydrate is occasionally ordered. It should not be larger than an ounce or an ounce and a half, and should be given with a glass syringe and tube as recommended for sedative enemata. Before it is administered the bowel should be cleared out by means of a purgative enema. Whenever such an enema is ordered it must be given without delay, because the effect of chloral hydrate upon the heart is to depress it, and necessarily this vital organ is very much weakened in a patient who is having a succession of fits, the weakening of the heart being a progressive process so long as the fits continue.

### The Nurses' Clinic.

### VAGINAL OVARIOTOMY CASES.

THE operation of ovariotomy for disease of one or both ovaries is sometimes done per vagina. The preparation of the patient for the above operation is as follows :—The parts must be shaved and the patient must have a warm bath. An aperient must be given about midday on the day previous to that of operation, and a soap and water enema, and antiseptic vaginal douche that same evening and the next morning. It is well to give a wash-out enema and vaginal douche again, if possible, within an hour before operation.

The patient must have a very light breakfast, not less than four hours previous to operation; she must pass urine immediately before going to the operating theatre, and must be warmly clad in a dressing-gown and long stockings, a blanket being spread over her body when on the operating chair. She will be operated on in the lithotomy position.

The surgeon will require the following instruments, etc. :—Irrigator (with warm antiseptic solution), douche nozzle, catheter, speculum, scalpel, vulsellum forceps, ovum forceps, artery forceps, dissecting forceps, pedicle needle, small curved needles, needle-holding forceps, ordinary straight or angular scissors, long uterine scissors, strong silk or catgut ligatures, plugs of iodoform gauze (for packing the vagina), swabs of sterilised gauze, pads of gamgee, antiseptic solution, sterilised towels, and T bandage.

When the patient is put back in bed she must be kept warm by hot-water bottles and blankets, her knees being supported by a pillow.

There will probably be a gradual oozing of hæmorrhage from the vagina for the first few hours after operation; the nurse must look from time to time, changing the pads, sponging the parts with warm water and drying thoroughly, drawing the sheet if necessary so as to keep the patient dry and comfortable. Unless the hæmorrhage becomes excessive there is no cause for alarm, and in that case the doctor in charge must be informed without delay.

The nurse is usually instructed by the surgeon to remove the vaginal plugs about twenty-four hours after the operation; if more than one plug has been used care must be taken that all have been removed. Vaginal douching is then commenced and usually continued, at least once daily, for from ten days to a fortnight.

The patient will probably be allowed to get up in less than a fortnight after the operation. Ordinary light diet may be given as soon as the patient has recovered from the anæsthetic sickness. An aperient is usually given on the first day after operation, and after that when necessary. Patients who have had this operation have sometimes a little difficulty in passing urine themselves for the first day or two; in this case the catheter may be necessary, but not oftener than eight hourly, and then with the utmost surgical cleanliness being observed on the part of the nurse.

### Incidents in a Nurse's Life.

### A PATIENT AT THE HOSPITAL FOR INVALID GENTLEWOMEN.

THE *fiat* had gone forth. I must undergo a serious operation, and that without loss of time. My doctor sent me up to a London surgeon, who told me to apply for admission to the Hospital for Invalid Gentlewomen in Harley Street. I did so, and, after a brief delay—having meantime found a substitute to take my place—I was told that there was a vacancy for me. Accompanied by a friend, I went to the hospital, where, on arrival, I was met by the matron with a most kind welcome, and was not even allowed to carry my handbag upstairs. I was introduced to my nurse and shown the room allotted to me. It was wonderful to me how all the horror and dread, which had been weighing me down ever since I had heard the verdict, seemed to slip from me never to return, as soon as I had set foot within the hospital. There was something in the air of the place which reassured me. My room was large, long, and airy, and situated at the top of the building. There was an additional window, looking on to the stairs, an arrangement I found occasion to bless, as I was thereby enabled to hear much of the service held in the cubicles below on Sunday evening.

My operation took place at 8.15 on the morning after my arrival, and for two days afterwards I was naturally rather miserable, and for about ten days underwent the—to me—

INCIDENTS IN A NURSE'S LIFE—*continued.*

entirely novel experience of utter helplessness. During all this time nothing could exceed the kindness and attention I met with on all sides. A bell was attached to me, which I was bidden to ring whenever I wanted anything, and it always seemed to me that the nurses must have been waiting outside the door, so promptly did they answer the summons, and always with a cheerful smile, as though pleased to do anything for me. The day after the operation my first visitor was admitted, for five minutes only; subsequently my friends were allowed to stay longer, and often had tea, which was provided for a small payment.

One of the Sundays while I was at Harley Street happened to be Hospital Sunday, and we received gifts of flowers and fruit from a neighbouring church, and were ourselves enabled to contribute to the Fund, as a collecting-box was brought round. There was in addition an early celebration once a week for any patients who were well enough. On my last Sunday I came down to the service, and was installed in an arm-chair, and forbidden with dreadful threats to stir from it till the close of the service. At last, after seventeen days, came the time for my departure, and I was truly sorry to leave all my kind friends, to whose care and attention I feel that I owed my speedy recovery. My time in the hospital, too, did me good in more ways than one, and was a real rest after the year's work, and also I am sure that my patients will benefit from my experience, as I learnt much I shall never forget by seeing the other side of the shield.

### The Cost of Catering in a Provincial Hospital.

BY A MATRON.

It is an interesting question whether the cost, generally speaking, of catering for a provincial hospital is greater than that for a London hospital.

For three years I catered in a London hospital of 100 beds, and for two and a half years I have been a matron in the provinces, and I find that I can provide better food at a smaller cost per head in the provinces than I could in London. The meat here is English and of good quality. In London we used foreign meat, also of good quality, but wanting in the flavour, which is found in English meat. In London nearly all goods were had at contract price. Here we contract for meat, bread, bacon, and milk only.

The following table shows the prices for meat compared to what we paid in London :—

| Provinces. *English.* | per lb. | London. *Foreign.* | per lb. |
|---|---|---|---|
| Mutton, all parts ... | 6¾d. | Mutton, leg ... ... | 5d. |
| Veal, all parts ... ... | 6¾d. | Mutton, shoulder ... | 4d. |
| Pork, all parts... ... | 6¾d. | Mutton, neck ... ... | 3d. |
| Beef, all parts ... ... | 6½d. | Beef, sirloin ... ... | 5d. |
| Beef-tea meat (shin without bone) ... | 4d. | Beef, steak ... ... | 6d. |
| Shin without bone ... | 4d. | Beef, shin, no bone | 3d. |
| Beefsteak ... ... ... | 6½d. | Pork ... ... ... ... | 6½d. |
| | | Veal ... ... ... ... | 7d. |

At this hospital the cost of meat per head for the year ending June, 1905, was £4 3s. 4d., and in the year ending June, 1906, it was £3 13s. 2d. In the year ending June, 1905, 68, and in the year ending June, 1906, 57 persons was the daily average. This includes patients and staff. I account for the decrease in the cost per head by the fact that the number of beds available has decreased, and the convalescence of the patients was consequently shortened owing to the pressure on the beds. Thus there were fewer patients on full diet. The meat allowance for patients is, men 4 oz. cooked, women 3 oz. cooked. This I carved and weighed myself daily.

The small average cost per head as compared to London hospitals is partly accounted for by the fact that in the London hospitals there is a larger staff of men to be fed—doctors, clerks, dispensers, porters. Here there is only one resident doctor and a dispenser, who has lunch twice a week. The porter lives in the lodge and has no food provided. The presents from friends of the hospital are a great help. In the year ending June, 1905, 40 rabbits and 80 pheasants were received, and in the year ending June, 1906, 121 rabbits and 59 pheasants were received. I think quite as many were received in the hospital in London where I catered, but I have not kept a record of the number.

With regard to butter, we have it from a farm, and for this we pay market price. It is excellent and all have the same. The following table shows the market prices for the year ending June, 1906 :—For 5 weeks, 11d. per lb; for 4 weeks, 1s. per lb. ; for 5 weeks, 1s. 1d. per lb. ; for 11 weeks, 1s. 2d. per lb. ; for 2 weeks, 1s. 3d. per lb. ; for 15 weeks, 1s. 4d. per lb. ; and for 10 weeks, 1s. 5d. per lb. The average cost per lb., 1s. 2⅜d. The cost of butter per head per week for the year ending June, 1906, was, therefore, 6⅛d. The allowance per patient of 6 oz. I do not always find sufficient, so that it is increased when necessary. The servants have an allowance of ½ lb. per head per week. For the nurses there is no allowance, and I find that they consume about 10 oz. per head per week. Very little butter is used for cooking; 1¼ lb. per week is the maximum. Lard and dripping are employed instead.

In London the contract price for butter was 1s. 2d. per lb. fresh, and 1s. per lb. salt. While I was in the metropolis I found the butter account most difficult to manage. The nurses used 14 oz. per head per week, and the cook used 7 and 8 lbs. a week for cooking purposes.

Potatoes are sent in large quantities from the harvest festivals in the neighbouring villages, so that the small sum of £5 4s. 7d. was all that was spent on potatoes in the year ending June 1906 as compared with £30 in the year ending June 1904. Vegetables and apples are also received from the churches; about one-fourth of what we use comes to us as gifts. As to eggs, we preserve them in water-glass, buying them when we can get twenty for a shilling. Including the water-glass the total cost is £4 7s. 8d. to preserve 1,600. When eggs are very dear we buy only a few new-laid for special use. We do not boil these preserved eggs, but only poach and fry them. In London the contract price for eggs was 1d. each. When eggs were plentiful they were fresh, and when eggs were dear they were not.

On first coming to this hospital I found the fish bill a difficulty. I could obtain very little fish under 7d. and 8d. per lb., and as the town is on a tidal river and only seven miles from the sea I thought this too much. The one fish-shop had a monopoly. In a short time I heard of a woman whose sons are fishermen, and she now brings fish daily, and charges 4d. per lb. for all the common varieties—cod, haik, whiting, lemon-soles. The fish is beautifully fresh, and it is frequently delivered alive.

For fowls we pay market price. This varies from 2s. 3d. to 3s., according to season, for fowls weighing 2¼ to 4 lb. each. In London we had a contract, and paid 2s. 9d. each.

Milk is 2½d. per quart in summer and 2¾d. in winter.

In London it was 2⅜d. in two-gallon cans, sealed, and 4d. per quart in sealed bottles.

The grocer's bill has been much lessened by the pound day collections from the villages and town. The following were received :—Tea, 100 lb.; sugar, 653 lb.; rice, 200 lb.; tapioca, 100 lb.; cornflour, 50 lb. The money saved in consequence I reckon at about £30. We pay more for groceries than in London. As we make our own jam and marmalade we save in this way. The average cost for making this is 3d. per lb. We pickle onions and red cabbage, and make marrow and apple chutney. We also make our own potted meat. I have been treated in a most generous way by my committee, who have allowed me to purchase goods that were not by contract where I thought best. In consequence of thus having a free hand I am able to study the provisioning in every light. By means of constant attention to detail, by personally supervising to see where waste occurs, and by the help of a good economical cook, I was enabled in my first year to reduce the total cost of provisions to £254 18s. 7d. less than the previous year. This, too, was with an increased staff of nurses and servants. There is a generous diet both for patients and staff, and we endeavour to vary the menu as much as possible, having no fixed days for certain things. The total cost of provisions per head per week is 4s. 7d. Subjoined is a three-days' diet-sheet for the nurses :—

TUESDAY.

*Day Nurses.*

Breakfast : Fried bacon, tea, bread-and-butter.
Lunch : Coffee, hot and cold milk, bread, cheese, beef-dripping.
Dinner : Roast beef, greens, potatoes, baroness pudding, cup of tea after.
Tea : Tea, bread-and-butter, marmalade.
Supper : Cold pork, cold mutton, beetroot, bread-and-butter, milk pudding, hot coffee, and cold milk.

*Night Nurses.*

Breakfast : Fried bacon, bread-and-butter, tea.
Night Meal : Soup, sardines, tea, bread-and-butter, jam.
Dinner : Chops, tomatoes, potatoes, cake pudding.

WEDNESDAY.

*Day Nurses.*

Breakfast : Kippers, bread-and-butter, tea.
Lunch : Coffee, hot and cold milk, bread-and-cheese, treacle.
Dinner : Steak pudding, haricot beans, potatoes, milk pudding, tea after.
Tea : Bread-and-butter, tea, and jam.
Supper : Soup, cold beef, pickles, bread-and-butter, hot coffee, cold milk.

*Night Nurses.*

Breakfast : Kippers, bread-and-butter, tea.
Night Meal : Eggs, porridge, bread-and-butter.
Tea : Marmalade, coffee.
Dinner : Sirloin of beef, greens, potatoes, boiled suet pudding and treacle.

THURSDAY.

*Day Nurses.*

Breakfast : Sausages, bread-and-butter, tea.
Lunch : Coffee, hot and cold milk, cheese, biscuits, bread.
Dinner : Roast mutton, greens, potatoes, jam tarts.
Tea : Bread-and-butter, jam.
Supper : Fish pie, cold beef, coffee, hot and cold milk, bread-and-butter.

*Night Nurses.*

Breakfast : Eggs.
Night Meal : Sausage-rolls, milk pudding, bread-and-butter, tea, coffee.
Dinner : Steak, greens, potatoes, baked batter pudding.

The above diet is for three days in winter. In summer eggs are more frequently given, no sausages, and in the hot weather cold meat and stewed fruit. The nurses have cake for tea on Sunday only.

### The Brooklyn Institute for Nurses and Nursing.

THE advent of the trams compelled Mrs. Cameron, the Superintendent, after nine years' residence in Anerley, to seek other quarters for her Institute, and shortly after Christmas patients and nurses took up their residence at their new abode, 2 Sydenham Avenue, Crystal Palace Park Road. The large house stands well back from the road in its own grounds. Originally a school, it is admirably adapted for its present purpose and affords accommodation for eight patients, the staff, such private nurses as may be temporarily resident, and servants. In the grounds are a tennis and croquet lawn and a poultry run, whilst there are plenty of quiet corners where patients may sit with the comfortable assurance that they cannot be overlooked. The dining-room has three French windows leading on to a balcony, and here too the patients can sit when they can come down, in restful chairs. Some of the patients' bedrooms are very commodious, others smaller, but all bright and pleasant-looking and of a good height. There are a small operating-room and a kitchen upstairs. The private nurses have a sitting-room which is in the school part of the house and consequently quite separate. Their boxes and chests of drawers are kept in what was the gymnasium, and the stock boxes in a cottage in the grounds. There are twenty-five fully trained nurses on Mrs. Cameron's books, and it is rarely that there are any in. The staff consists of the home sister, a charge nurse, a night nurse, and a probationer. Accommodation for nine private nurses is always ready in the Institute, but there are two emergency beds in case of necessity. The Brooklyn Institute has been full ever since the move.

### Infants' Weight Chart.

(W. H. BAILEY AND SON, LIMITED, 38 OXFORD STREET, W.)

THIS is a useful chart for recording graphically a child's weight each week during the first year of life and comparing it with the average weight for the same week. Most bottle-fed babies suffer for a time from more or less flatulence and slight digestive disturbances, although really doing well and gaining weight. In such cases the chart may bring solace to the troubled mind of a mother over-anxious about her first-born; for, sad to say, the frequency with which infants are regularly weighed usually varies inversely with the number of older children in the family. On the other hand, a fall in weight to below the average for the child's age may in good time direct attention to the fact that his food is inadequate, although he may apparently be thriving. Judging from its size, the chart is intended to be fastened to the nursery wall, for if a piece of paper so large and thin is left about the room the child may survive the perils of the first year but the chart probably will not.

### Illustrations of the Life of a Modern Nurse.

LIFE IN A FEVER HOSPITAL.

ON DAY DUTY.

A WARD AT THE PARK HOSPITAL, HITHER GREEN.

### Dr. Hutchison on Infant Feeding.

A LARGE audience, of whom a great many were nurses, gathered at the Institute of Hygiene on Wednesday last week to listen to a lecture on "Infant Feeding" by Dr. Robert Hutchison, M.D., F.R.C.P. After dwelling on the importance of natural feeding wherever it was possible, Dr. Hutchison proceeded to discuss the different methods of artificial feeding that might be followed. When cow's milk is used for a child he recommended its dilution with not more than an equal quantity of water. It is true that with some children this mixture does not agree, but he did not believe that better results were to be got by a greater dilution, which only made the milk too watery without getting over the difficulty of getting rid of the superfluous casein, the presence of which could always be detected by the child having sickness, colic, and unhealthy motions. Dr. Hutchison suggested various expedients for increasing the digestibility of cows' milk in cases where the half-and-half mixture did not give satisfactory results.

1. CITRATED MILK—*i.e.*, the addition of citrate of soda, which is a harmless vegetable salt, to the food in the proportion of 1 grain of the soda to 1 oz. of milk—that is, to 2 oz. of the half-and-half mixture. He admitted, however, that there were certain objections to the use of the salt, and that it was best to employ it only under the direction of a medical man.

2. CONDENSED MILK.—If the citrated milk failed to give satisfactory results, it would be necessary to fall back on some good brand of condensed milk, of which there are several on the market. He recommended the sweetened preparations, as the unsweetened do not keep after the tin has been opened. He also advised his hearers to disregard entirely all the directions on the tins. As to the proportions to be used, he recommended a mixture of one teaspoonful of milk to six tablespoonsful of water. This mixture was almost right as to the proportion of casein, but was comparatively poor in fat, but the addition of a teaspoonful of cream would make a combination that would be something very like human milk. While the preparation would in most cases give satisfactory results, the use of it should not be continued too long. The outside limits would be the age of four months, and if this kind of feeding was prolonged there was a risk of the child developing rickets.

3. DESICCATED MILK.—This is a form of milk that is not yet well known, but Dr. Hutchison predicts a future of great usefulness for it. It is at present difficult to obtain, but is both convenient and valuable. Many children can digest a good deal of desiccated milk, when the same quantity of cow's milk would disagree.

4. PEPTONISED MILK.—Where the child is so delicate that none of these forms of milk can be digested, one must fall back on peptonised milk, the pepsin being added to the half-and-half mixture. But Dr. Hutchison warned his hearers that if a child was sufficiently ill to need this kind of food, it was sufficiently ill to require the care of a doctor.

With regard to the time of feeding, he gave the following table :—

TIME-TABLE FOR FEEDING.

| Age. | Intervals by Day. Hours | Number of Night Feeds. | Approximate Quantity at Each Feed. Ozs. |
|---|---|---|---|
| 1 week | 2 | 2 | 1 to 1½ |
| 2 to 3 weeks | 2 | 2 | 1½ to 3 |
| 4 to 5 ,, | 2 | 1 | 2½ to 3½ |
| 6 to 12 ,, | 2½ | 1 | 3 to 4½ |
| 3 to 5 months | 3 | 1 | 4 to 5½ |
| 5 to 9 ,, | 3 | — | 5½ to 7 |
| 9 to 12 ,, | 3½ | — | 7½ to 9 |

As to the amount to be taken at any one meal, he advised that this matter should be left to the baby. The appetites of adults were not all alike, and neither were those of infants. He also advised a simple oval bottle, with a plain rubber teat, and deprecated the putting of too much trust on valves and other contrivances to regulate the pressure. It is important, however, to see that the hole in the top of the teat is big enough, and not too big. If it is too large the child is fed too fast; if it is too small the baby has to exhaust itself in sucking. A safe test is that if, when the bottle is held upside down, the milk drops out at the rate of a drop per second, the hole is the right size.

### A Middlesex Hospital Nurse and the Jamaica Earthquake.

MISS S. CROSS, a nurse attached to the private staff of the Middlesex Hospital, who went out to Jamaica in the *Port Kingston* with a patient, was able to render the most valuable service in nursing the sufferers from the earthquake who were conveyed for treatment on to the boat, which, for the time being, was converted into a floating hospital.

To our representative she described the scene on arriving on board as one of fearful confusion. "The injured were strewn all over the decks, some already dead, the crew running to and fro to find mattresses and rugs. Another bargeful arrived a little later, but the officer called Dr. Evans down to see them before he unloaded, and several were dead, so those were taken back to the shore." Asked if she saw anything of the Mildmay nurses, or any other doctors or nurses, she said "No, they were busy on shore, and Lady Swettenham was helping them, and we had not a minute to spare after we got to work. Most of the injuries were terrible, and the anæsthetics gave out long before the operations were finished. We gave them all an injection of morphia afterwards, and one woman whose face was cut open, and her eye actually laid out on her cheek, bore the sewing up with no anæsthetic without a murmur, but as soon as she saw the hypodermic needle she shrieked with terror."

Miss Cross found the officers and crew most helpful, and says that they worked all night too. In fact, no one got much rest till after the boat left the harbour. The last day was a very busy one, as all the patients had to be moved from the vessel to the sheds which had been temporarily erected on the banana wharf. They were laid on a mattress placed on a shutter, which was then raised at the four corners by the crane, and lowered into the barge alongside. Eight of the injured were among the passengers, and so had to be nursed on the homeward voyage, besides one girl, who suffered from typhoid. One patient had received a severe injury to the knee. A little boy had both legs broken, and as his father and mother had lost everything in the fall and burning of their house, and were penniless, Sir Alfred Jones brought them home to their relations in England. The mother was injured in the shoulder by a falling beam, but the injury was not observed for a day or two till suppuration commenced. This happened in several otherwise minor cases, in consequence of the dirt being so ground in by the weight of the falling timber. The medical stores naturally ran very short on board, but fortunately there was a lot of carbolized tow among the ship's goods, so the splints were padded with that, and the sheets were torn up for bandages.

At present Miss Cross is resting, and the matron of the Middlesex Hospital, says that until she is eating and sleeping well and her nerves are thoroughly restored she will not resume her nursing work.

### The Royal Infirmary of Edinburgh.

### RESIGNATION OF THE LADY SUPERINTENDENT.

Miss Spencer, having intimated her intention of relinquishing her duties at June 30 next, the managers have minuted their appreciation of her services and their regret at her retirement in the following terms :—

"The managers have received with much regret the letter of Miss Spencer, Lady Superintendent of Nurses, announcing her intention of retiring from her post on June 30 next, on which date she will have completed thirty years of service in the Royal Infirmary, and they desire to place on record their high appreciation of her work during this long period.

"Entering the service of the Royal Infirmary in 1877 as assistant to Miss Pringle, the then Lady Superintendent, Miss Spencer during ten years discharged her duties with such conspicuous ability and aptitude that in 1887 she was unanimously chosen by the managers to succeed Miss Pringle, who had received another appointment. Throughout the whole twenty years Miss Spencer has held the office she has given such unwearied, devoted, and whole-hearted attention to its duties as to call forth the admiration of all who have had opportunity of observing and knowing the amount and character of the work she has had to do. During this period, which has witnessed a large extension of the infirmary, the manner in which Miss Spencer, in the midst of her difficult and responsible duties, has met the increasing demands upon the nursing and domestic arrangements has been such as none but one specially gifted for the work could have done.

"Recognising as they do that the reputation and success of the Royal Infirmary as a house of healing for the sick and suffering depends in a large measure upon the equipment and efficiency of its nursing staff, the managers have great satisfaction in recording their warm appreciation of Miss Spencer's unceasing efforts by careful selection, by improved methods of training, and by attention to all that pertains to the comfort and well-being of the nurses, to raise this department of the infirmary's work to the high position it now holds. Further, the managers have had, during all Miss Spencer's period of service, the gratification of observing the high tone which pervades the whole department of which she has the oversight. They feel that this is eminently due to the influence of her personal Christian character and example, her wise supervision, and to the lofty ideals of the noble and sacred calling of a nurse which she has at all times striven to set before those who were, or came to be, under her charge.

"The managers trust that the sorrow Miss Spencer cannot fail to feel at leaving the work so near to her heart may be mitigated by the knowledge that she has been privileged to give her best years to a calling and a cause worthy of life's highest ambitions and energies; and that she carries with her the affectionate regard not only of the present nursing staff, but of many hundreds of nurses in all parts of the world who have been trained under her care. The managers assure Miss Spencer of their genuine gratitude, their high esteem, and their cordial wishes that all good may attend her in the future.

### Presentations.

Barmouth Nursing Association.—The Committee of the Barmouth and District Nursing Association have presented a gold watch to their Queen's Nurse, Miss Elizabeth A. Jones, who has carried on the work in the district for ten years (being the first nurse), in appreciation of her excellent work.

### Everybody's Opinion.

### A PROBATIONER LOSES HER SIGHT.

"Policy 192, Royal National Pension Fund for Nurses," writes : Please give the half-crown enclosed to the probationer of West Ham Infirmary who lost the sight of one eye. I sincerely sympathise with her.

Nurse Ruth, Glasgow, writes : May I ask you to add the enclosed small contribution of 15s. to the fund for the nurse who recently lost her eyesight while she was in attendance on a "typhoid" patient at West Ham Infirmary? If anything can comfort her for the loss of the work she loves it will be the thought that she did her duty unflinchingly, and I should like to offer her the assurance of my respect as that of a sister-nurse who hopes that she would be able to forget "self" as generously under similar circumstances.

### NURSES AND THE WORKMEN'S COMPENSATION ACT, 1906.

Mr. S. Buchanan Smith, Secretary, The Infirmary, Salisbury, writes : There seems to be some doubt whether the provisions of the above Act would apply to the nurses of a hospital, and the committee of this infirmary would be glad to know the views of the officials of London and other hospitals on the matter. May I therefore ask the favour of the question being ventilated in The Hospital with the object of ascertaining the general opinion as to whether the authorities would be liable under the above Act in case of accident, fatal or otherwise, to nurses (paid and unpaid) while in their employ?

[There can be little or no doubt that hospital nurses, including probationers, come within the definition of "a person who . . . works under a contract of service or apprenticeship with an employer." So long, therefore, as a nurse's remuneration is less than £250 a year she is entitled to claim the benefits conferred by the Act. It should, however, be remembered that her employers would only be liable for "accidents arising out of or in the course of her employment," and that this would not (in the case of a hospital nurse) include *diseases* contracted while nursing, unless directly attributable to a previous accident, such as a cut finger resulting in blood-poisoning. The risk incurred by hospital authorities is one that they should certainly insure against.—Ed. The Hospital.]

### NURSING SPOTTED FEVER.

"Interested" writes : I was very interested in reading the description of spotted fever in Belfast in the last issue of the *Nursing Mirror*, as I nursed a case on the district this last summer diagnosed meningitis, but about which the doctor was doubtful, as the symptoms were like those described in "spotted fever." I was called in on September 22, as the boy (who was four years old) had fallen off the form at school and vomited—the vomit being described as red, which was put down to his eating red berries, but this was not proved. Hæmorrhagic spots appeared all over the body in patches on the second day, and came out in fresh crops three times within a week. The skin in parts gradually peeled off, and in two or three places formed an ulcer. The child grew thinner every day and screamed at every movement. The vomiting continued for over two weeks, and was an almost black fluid. Nourishment was taken well for a day or two at a time, and then for one or two days refused, when the temperature dropped, but fluctuated for several weeks. I paid 102 visits in all, and the boy was convalescent at the end of November. He was put on a water bed, washed, hips and back rubbed and anointed twice daily, and latterly rubbed gently all over with olive oil. On November 3rd he was put into a fresh room with entirely fresh clothing, bed, etc., and from that time began to recover. For a fortnight he appeared to be paralysed, but he very gradually gained the use of his limbs and muscles. He had "Kepler" solution of malt and cod liver oil for several weeks during convalescence, and, though now not looking over-robust, he is running about. No one else took the disease, though a great many friends and neighbours went to look at him and assisted with night duty, and no disinfecting was done, except by air—the window being open night and day.

### THE DISTRICT NURSE AND THE PARISH DOCTOR.

"E. W." writes: I should feel grateful if you will print this for me in THE NURSING MIRROR, as I should like the opinion of others whether I did right under the following conditions: I am working in Cheshire as a district nurse. The district nurse depends upon her cases being sent in by the doctors, so that if she offends they give her no work. That has happened several times to other nurses before I came. The parish doctor here is rather difficult to work under, but as he is the nurses' doctor as well I have managed for a year and a half not to offend him. Lately I was taken ill with influenza. He met me at a patient's house. I was then very unwell, and he just said, "Work it off, like I have to do." Next day I had to stay in bed, and was so ill that I sent for him. He never came, and several ladies on the committee wanted me to send for another doctor, but I told them that I would wait until next day. He did not come then, so I sent for another doctor, who came, and was most kind. The parish doctor turned up twenty-four hours after, so I told him I had another doctor. He said that I ought to have sent again for him, and one lady on the committee thinks the same, and that I did very wrong. Now I am told that the parish doctor will not give me any cases. It seems very hard to be so restricted.

### THE CONSUMPTIVE AT HOME.

"MIDGET" writes: The article appearing in your number of February 2 interests me greatly, having had a large experience of work in sanatoria, and the difficulties in providing the after-treatment which is often so necessary, for the well-being of the patient and the safety of his friends. For people with plenty of money it is generally easy to persuade them that the treatment must be continued, and the life led a healthy one, but for the patient with his living to earn this further treatment becomes a far greater problem than most people imagine. In some sanatoria every effort is made to provide them with healthy outdoor exercise in the case of men, or in the case of women with an indoor life under the most favourable circumstances. Where they live in towns they are advised to remove to the country if possible, but here want of means often comes in the way. Many of the patients have spent their savings to secure the open-air treatment, without which they would probably have died, and frequently they have to return to work long before they are fit, as no more money is forthcoming. Some have fairly comfortable homes where friends willingly assist them to carry on the treatment, and one is often cheered by the favourable reports sent in from time to time. But many have no homes to which they can return; they have to go to lodgings and manage as best they can. It is surprising sometimes how much is done by the persevering ones; many of them change their work, though with great difficulty. For instance, it is by no means easy for a clerk to find outdoor occupation, which he is able to do, or for a woman to obtain the suitable indoor employment. Some are unable to change their work, and must return to their former life, and it is sad to feel that they must almost inevitably fall ill again. Others, of course, have their homes with gardens, where a father or brother or friend gladly builds a shelter in which the patient spends his time quite cheerfully, making steady progress, defying the weather under any circumstances, and becoming a wonder to all his friends, and to whom he gives faith in the fresh air and the good, wholesome food which he knows is life to him. It is a matter of regret that so few nurses have any practical experience of sanatoria and their methods. People are always influenced by those who speak of a subject of which they are master. Frequently a nurse comes in contact with people in the first stage. She sees at once medical advice is necessary, so that the doctor may not have to say later, "If only I had seen this patient before how much might have been done." But sometimes it is very difficult to persuade people who only feel a little ill to consult a medical man. Then the nurse might impress a few simple things on the invalid, the need for fresh air, the care of sputum, and the danger to other members of their family. In this way much can be done to prevent the disease spreading. I have heard nurses say that sanatorium work seems wasted time. Why? because they want to do something great. I quite agree that it is often much more difficult than hospital work where the cases are so varied, and at times one gets very discouraged, as the patients are a long time before they make much progress, but anyone taking up the work with the spirit of doing what little they can to fight against consumption will be amply repaid.

### Appointments.

BELPER WORKHOUSE INFIRMARY.—Miss Kate D. M. Underwood has been appointed superintendent nurse. She was trained at Birmingham Infirmary, where she has since been night sister.

DEVONPORT WORKHOUSE INFIRMARY.—Miss Lillie Edworthy has been appointed charge nurse. She was trained at Nottingham Workhouse Infirmary, and has been children's attendant at King's Norton Union, Birmingham.

ROYAL HOSPITAL FOR SICK CHILDREN, EDINBURGH.—Miss Isabel Bothwell and Miss Mabel Cooper have been appointed staff nurses. Miss Bothwell was trained at Pendlebury Children's Hospital, and has since been charge nurse at the Manchester Children's Hospital Convalescent Home. Miss Cooper was trained at Bradford Children's Hospital.

ROYAL UNITED HOSPITAL, BATH.—Miss Ida Nash has been appointed sister of the women's medical wards. She was trained at the Royal United Hospital, Bath, where she was afterwards on the private staff and night sister. She has since been sister at the Hertford British Hospital, Levallois Perrett, Paris.

STOCKPORT INFIRMARY.—Miss G. Fitzgerald has been appointed sister. She was trained at Manchester Royal Infirmary, where she has since been staff nurse. She has also been night sister at Grantham General Hospital.

STROUD HOSPITAL.—Miss Annie Charteris has been appointed charge nurse. She was trained at the Royal Infirmary, Dumfries, and has since been ward sister and night sister at Burnley Hospital, ward sister and theatre sister at the East Sussex Hospital, and night sister at Chalmers' Hospital, Edinburgh.

THE POLYCLINIC HOSPITAL, PHILADELPHIA, U.S.A.—Miss Elsie Macdonald has been appointed matron of the Nurses' Home. She was trained at the Royal Infirmary, Manchester, and at East Pilton Fever Hospital, Leith, N.B. She has since been staff nurse at the Royal Infirmary, Manchester.

WATFORD ISOLATION HOSPITAL.—Miss Gertrude Praegh has been appointed charge nurse. She was trained at University College Hospital, London, and has since been charge nurse at the Eastern, Northern, and Western Hospitals under the Metropolitan Asylums Board, and charge nurse of the diphtheria wards in an Isolation Hospital. She has also done private nursing at Leicester.

### Queen Victoria's Jubilee Institute for Nurses.

MISS ANNE HISCOCKS has been transferred to Ashby-de-la-Zouch from Kilburn, and Miss S. Sullivan to Bolsover from Birmingham. Miss Barbara Lendrum has been appointed to Plaistow Maternity Charity and District Nurses' Home, and Miss L. E. Fenton to Huddersfield. Miss Marguerite Dancey has been appointed temporarily to Hackney, and Miss Selina Sellers also temporarily to Bath.

### The London Association of Nurses.

On Shrove Tuesday, February 12, an "at home" was held at 3 Burwood Place, under the auspices of the London Association of Nurses, when Mr. Louis Dick, secretary of the Royal National Pension Fund for Nurses, gave an address, explaining the advantages of the Fund, and urging membership on those who had not yet joined. Considering the busy time, a goodly number of nurses managed to be present, and the gathering was much enjoyed, though all were saddened by the news that within the last few days two promising young nurses on the staff of the Association—Miss Ross Rowley and Miss S. J. Glossop—had passed away.

### The Sanitary Inspectors Examination Board.

The following is a list of the candidates who passed in the examination for sanitary inspectors under the Public Health (Lond.) Act, 1891, held in January 1907: Miss C. Beeny, Miss E. Brown, Miss F. E. Evans, Miss M. D. Herskind, Miss E. G. M. Johnson, Miss C. E. Moir, Miss Moor, Miss E. B. Taylor, Miss J. C. Wienholt, Miss M. G. Williams received instruction from the National Health Society; Miss Elliott, Miss J. A. Jacobs, Miss J. J. Rawlings, Mr. J. D. Saul, Miss G. Stevens, received instructions from the Royal Sanitary Institute; and Miss H. Bhose received instruction in Office as Inspector.

### Central Midwives Board.

### EXAMINATION PAPER.

At the examination of the C.M.B. on Tuesday the following questions were put to the candidates:—

1. Describe the normal appearance of the placenta and membranes immediately after delivery at term. What is a succenturiate placenta, and what is its importance?
2. You are called to attend a multipara at full term who has been for two hours in labour. She tells you that the waters have just broken. Describe in detail the examination you would think it necessary to make.
3. What is the nature of after-pains? Under what circumstances do they occur, and how would you recognise and treat them?
4. Describe in detail the management of twin labour after the birth of the first child, and give reasons for all you do.
5. What abnormal appearances of the skin may be met with in the first ten days of infant life? How do you distinguish them from one another?
6. What are the duties of the midwife, according to the Rules of the Central Midwives Board, towards the patient in regard to the following points?—(*a*) In the matter of staying with the patient after labour has begun; (*b*) passing the catheter; (*c*) if the life of the new-born child appears to be in danger.

### Queen Alexandra's Imperial Military Nursing Service.

Miss E. McGrath, Miss C. M. MacRae, Miss M. H. Smyth, and Miss M. C. Watson have been appointed staff nurses in Queen Alexandra's Imperial Military Nursing Service. Miss A. C. Mowat, staff nurse, has been transferred to the Military Hospital, Gosport, from the Queen Alexandra Military Hospital, Millbank; and Miss H. Winzer, to the Queen Alexandra Military Hospital, Millbank, from the Military Hospital, Gosport.

### Death in our Ranks.

On Saturday last there passed away Nurse Maude Kathleen Hutchinson, aged 27, late of Lambeth Infirmary, after 19 weeks' illness, caused by "ulcerative endocarditis" and "septic peritonitis," contracted whilst nursing. Her sufferings were borne with patience and fortitude. She was laid to rest on Monday in the parish churchyard of St. Mary Wolborough, Newton Abbot, Devon.

### Where to Go.

"Gardens of Delight."—Under this title Mrs. Cardwell Crofton is exhibiting a number of her water-colour pictures of gardens at the Modern Gallery, 61 New Bond Street, W., until February 26. This is the second exhibition Mrs. Crofton has given, and the 68 pictures form a delightful gallery of beautiful gardens of every type, which cannot fail to give pleasure to all lovers of nature and the country. Mrs. Crofton possesses the power of giving the atmosphere of gardens, and her paintings of flowers and trees is quite remarkable for its excellence.

### Novelties for Nurses.

### A TESTIMONIAL BOOK.

Many nurses must have experienced the discomfort of carrying about with them frequently "somewhere at the bottom of their box," where they become crushed and dirty, such testimonials as they have received and which they really highly value. A neat little book has just been produced by the Nurses' Co-operative Association, Stockport, which should make a welcome and useful private gift from one nurse friend to another. It has crimson-padded covers and gilt edges for 2s. 6d., or it may be obtained in plainer style for a shilling. The doctor, matron, or patient who is anxious to record pleasant things of the owner can be asked to write in the book direct instead of on a loose sheet of paper, and it is safe to prophecy that this dainty "Testimonial Book" will soon be ranked amongst the most necessary possessions of many nurses. There is some valuable information for reference on the first few pages, including an obstetric table, medical baths in common use, and general rules of quarantine.

### A COMPETITION FOR CHRISTMAS, 1907.

The success of our experiment last year in inviting nurses to write our Christmas Supplement induces us to afford them the opportunity next year of contributing to our columns a special short story bearing upon hospital, infirmary, mental asylum, or district or private nursing life, not necessarily consisting of actual facts, but, if possible, founded upon them. The length of the story, which must, of course, be interesting and probable from first to last, should be about 5,000 words, and, other things being equal, preference will be given to the contribution which is accompanied by two or three good photographs or drawings, for purposes of illustration. The sum offered for the story is £5 5s., and the competition is open to nurses in all parts of the world. In order to stimulate effort, we shall send a cheque for £2 2s. to the author of the story which we consider second best, reserving the right to publish it. With the view of enabling our readers in the Far East and other distant quarters of the globe ample time to forward MS., contributions will be received up to June 30, 1907; but we shall be glad to have them as soon as convenient. They should be addressed to the Editor, Nursing Section of The Hospital, and marked outside "Christmas Competition."

### Notes and Queries.

**REGULATIONS.**

**The Editor is always willing to answer in this column, without any fee, all reasonable questions, as soon as possible.**

**But the following rules must be carefully observed.**

1. **Every communication must be accompanied by the name and address of the writer.**
2. **The question must always bear upon nursing, directly or indirectly.**

**If an answer is required by letter a fee of half-a-crown must be enclosed with the note containing the inquiry.**

*Training in Birmingham.*

(201) Is there a hospital in or near Birmingham where I could become a probationer and receive a small salary?—*Redditch.*

At the Infirmary, Birmingham, you can be received between 21 and 35 years of age at a salary of £10 the first year. But you must pay a premium of £20. At the Birmingham and Midland Hospital for Women, salary, first year, £8. We advise you to get and study "How to Become a Nurse," 2s. 4d. post free, from The Scientific Press, 28 and 29 Southampton Street, Strand, London, W.C.

*Children's Nurse.*

(202) I wish to become a children's nurse. Where can I train?—*Cranleigh.*

The Norland Institute, 10 Pembroke Square, W.; the Liverpool Ladies Sanitary Institute, 27 Leece Street, Liverpool; School of Domestic Science, Belmont House, Cheltenham, etc.

(203) Where can I train as a children's nurse?—*Glasgow.*

See answer to *Cranleigh.*

*Home for Old Gentleman.*

(204) I am anxious to find a home for an old gentleman. He could pay about 10s. weekly. Can you help me?—*Anxious.*

Apply to Sutton's Hospital, Charterhouse, E.C. There may possibly be a vacancy.

*Nursing Abroad.*

(205) Where can I apply for a post as nurse abroad?—*Brighton.*

There is a list of nursing homes abroad in "How to Become a Nurse," price 2s. 4d. post free, The Scientific Press, 28 and 29 Southampton Street, Strand, London, W.C., but we fear that you are too late for this season.

*Home for Old Woman.*

(206) Is there any home which would receive a helpless old woman?—*Southwark.*

Try Nazareth House, Hammersmith.

*Training in Sheffield.*

(207) I should like to become a probationer, with salary, in Sheffield. I am nearly 21.—*Sheffield.*

Apply at once to the Sheffield Workhouse, the Clerk to the Guardians, Union Offices, Westbar, and say you wish to put your name down at such a date as you will be 21. The salary is £10 the first year. Then try and educate yourself in the meanwhile. Improve your writing, and ask advice of some educated friend as to what you should do.

*Training in Nottingham.*

(208) Is there an institution in or near Nottingham where probationers receive a salary the first year?—*Nottingham.*

At the Nottingham General Hospital probationers receive a salary of £6 the first year, but they have to pay a premium of £10 10s. on entrance. The Children's Hospital, Nottingham, £5 first year; Nottingham Hospital for Women, £6; and Samaritan Hospital, £10; but only two years' training is given.

*Home for a Girl.*

(209) Can you tell me of a home for a girl of 22 who is not insane, but occasionally unmanageable? A small sum can be paid.—*A. H. L.*

Possibly the Cavendish Industrial Home, Pond Street, Hampstead, may suit. The charge is 5s. weekly.

**Handbooks for Nurses.**

| | Post Free. |
|---|---|
| "How to Become a Nurse: How and Where to Train." | 2s. 4d. |
| "Nursing: its Theory and Practice." (Lewis.) ... | 3s. 6d. |
| "Complete Handbook of Midwifery." (Watson.) ... | 6s. 4d. |
| "Preparation for Operation in Private Houses." ... | 0s. 6d. |
| "The Nurses' Enquire Within." ... ... ... ... | 2s. 3d. |
| "Nurses' Pronouncing Dictionary of Medical Terms" | 2s. 0d. |

Of all booksellers or of The Scientific Press, Limited, 28 & 29 Southampton Street, Strand, London, W.C.

### For Reading to the Sick.

THINGS OF BEAUTY.

A THING of beauty is a joy for ever:
Its loveliness increases; it will never
Pass into nothingness; but still will keep
A bower quiet for us, and a sleep
Full of sweet dreams, and health, and quiet breathing,

. . . . . . .

Such the sun, the moon,
Trees old and young, sprouting a shady boon
For simple sheep; and such are daffodils
With the green world they live in; and clear rills
That for themselves a cooling covert make
'Gainst the hot season;

. . . . . . .

Glories infinite,
Haunt us till they become a cheering light
Unto our souls, and bound to us so fast,
That, whether there be shine, or gloom o'ercast,
They always must be with us, or we die.

*Keats.*

The world of Nature tells us of the God with Whom we have to do. The book is always open, in which we can read of God's wisdom, power, and goodness. We learn of Him by the marvels of the earth and sea and sky. As we search more deeply, with more trained skill, into the things around us, we find ourselves face to face with ever new mysteries, which humble us as we vainly try to read them. God lets us see His work; but He does not let us watch Him at work, or know how and why things are as we find them.

The Father of lights, "with whom is no variableness, neither shadow of turning," gave being to the heavenly bodies, and gave them power to shed blessing on our world. He guides their courses, and rules the movements of the earth, so that it may more fully receive their influence. We need the bright days, and the warmth that beams from the cheering sun. But these would not be the blessing that they are, did not clouds sometimes darken over us. Those very clouds, indeed, are among the blessings that we owe to the sun. The frosts and snows and storms have their good work which God has given them. We reap in harvest a blessing, which the dark chill winter and the glowing summer have each shared in bringing to us.

We can trace a steady course in the world of Nature. So we know how to act, and what to expect. Sometimes strange things happen, which man has not yet learned to put into their place with other things. But we are sure that nothing goes from under God's control, and that, if we knew more, we should see that there is no breaking in on the order which God has fixed. In the world of grace we can mark the working of the same God; and we must look to find ourselves at fault, when we try to search beyond where God gives light to see. But we learn to trust, and to own humbly, that we cannot judge the power or wisdom of the God of grace. There is order where to us all seems confusion. Storms bring a purer air, and a more lasting calm. What seems to threaten ruin is needful to save from ruin, and to work salvation. Christ is the Sun of righteousness; and from Him grace and truth come to us, even though our sky is overcast.—*Anon.*

## Figures and Tables

**Figure f1:**